# Sex-specific differences in left ventricular mass and myocardial energetic efficiency in non-diabetic, pre-diabetic and newly diagnosed type 2 diabetic subjects

**DOI:** 10.1186/s12933-021-01248-z

**Published:** 2021-03-06

**Authors:** Elena Succurro, Sofia Miceli, Teresa Vanessa Fiorentino, Angela Sciacqua, Maria Perticone, Francesco Andreozzi, Giorgio Sesti

**Affiliations:** 1 Department of Medical and Surgical Sciences, University Magna Graecia of Catanzaro, Viale Europa, 88100 Catanzaro, Italy; 2 Department of Clinical and Molecular Medicine, University of Rome-Sapienza, 00189 Rome, Italy

**Keywords:** Sex-differences, Cardiovascular disease, Left ventricular mass, Myocardial mechano-energetic efficiency, Prediabetes, Type 2 diabetes

## Abstract

**Background:**

Women with type 2 diabetes (T2DM) have a higher excess risk for cardiovascular disease (CVD) than their male counterparts. However, whether the risk for CVD is higher in prediabetic women than men is still debated. We aimed to determine whether sex-related differences exist in left ventricular mass index (LVMI), and myocardial mechano-energetic efficiency (MEEi) in with normal glucose tolerant (NGT), pre-diabetic and newly diagnosed type 2 diabetic subjects.

**Methods:**

Sex-related differences in LVMI and myocardial MEEi, assessed by validated echocardiography-derived measures, were examined among 1562 adults with NGT, prediabetes, and newly diagnosed T2DM, defined according to fasting glucose, 2-h post-load glucose, or HbA1c.

**Results:**

Worsening of glucose tolerance in both men and women was associated with an increase in age-adjusted LVMI and myocardial MEEi. Women with newly diagnosed T2DM exhibited greater relative differences in LVMI and myocardial MEEi than diabetic men when compared with their NGT counterparts. Prediabetic women exhibited greater relative differences in myocardial MEEi, but not in LVMI, than prediabetic men when compared with their NGT counterparts. The statistical test for interaction between sex and glucose tolerance on both LVMI (P < 0.0001), and myocardial MEEi (P < 0.0001) was significant suggesting a sex-specific association.

**Conclusions:**

Left ventricle is subject to maladaptive changes with worsening of glucose tolerance, especially in women with newly diagnosed T2DM. The sex-specific increase in LVM and decrease in MEEi, both being predictors of CVD, may have a role in explaining the stronger impact of T2DM on the excess risk of CVD in women than in men.

## Background

Several cross-sectional and longitudinal studies as well as large-scale collaborative meta-analyses of prospective studies have shown that women with type 2 diabetes mellitus (T2DM) have a higher relative risk (RR) for fatal and non-fatal cardiovascular events as compared with men [1–7]. Although a greater burden of cardiovascular risk factors has been also observed in prediabetic women as compared with their male counterparts [10, 11], most of prior studies did not report higher RR for CVD in prediabetic women as compared with their male counterparts [8, 9, 12–16]. Thus, the question of whether the RR for CVD is higher in prediabetic women as compared with men is a subject of debate. A possible explanation for these disparities is that occurrence of major cardiovascular events, including myocardial infarction, and stroke, in women requires an exposure to higher levels of glycemia than those typically associated with the prediabetic condition. In this scenario, it may be useful to determine whether prediabetic women exhibit a greater cardiovascular organ damage as compared with their male counterparts, and whether the impact of the hyperglycemic burden begins to increase to a clinically relevant extent before exceeding the diagnostic threshold of T2DM. To this purpose, we took advantage of the opportunity to study a well characterized cohort of participants in the CATAMERI study, an ongoing observational study recruiting adult individuals with one or more cardio-metabolic risk factors who underwent a complete clinical characterization including an oral glucose tolerance test (OGTT), and HbA1c assessment, and standard Doppler echocardiography [17–19]. Specific aims of this study were to determine whether sex-related differences exist in left ventricular mass, and myocardial mechano-energetic efficiency, both independent predictors of cardiovascular events [20–22], among individuals with normal glucose tolerance (NGT), prediabetes, and newly diagnosed, defined according to all three American Diabetes Association (ADA) criteria, i.e., FPG, 2-h post-load glucose, or HbA1c [23].

## Methods

### Study participants

The study cohort comprised 1562 subjects participating in the CATAMERI study, an ongoing observational study recruiting adult individuals with one or more cardio-metabolic risk factors recruited at a referral university hospital of the University “Magna Graecia” of Catanzaro [17–19, 24]. The study subjects were recruited according to the following inclusion criteria: age between 40 and 70 years, and positivity for one or more cardio-metabolic risk factors including family history of diabetes, dysglycemia, hypertension, dyslipidemia, and overweight/obesity. Exclusion criteria included: previous diagnosis of type 1 or type 2 diabetes, established cardiovascular disease on the basis of medical history and resting electrocardiogram, uncontrolled hypertension, valvular heart disease, history of malignant or autoimmune diseases, acute and chronic infections, end-stage renal disease, liver cirrhosis, history of alcohol or drug abuse, glucose-lowering agents including metformin, and treatment with medicaments known to affect glucose metabolism such as corticosteroids and estroprogestins used for hormonal contraception or replacement treatment, or medicaments affecting myocardium workload including beta blockers and antiarrhythmic drugs. Eligible subjects underwent anthropometrical evaluation including measurements of body mass index (BMI), and waist circumference, blood pressure, and biochemical determinations. After an overnight fasting, a 75 g OGTT was performed in individuals with FPG < 126 mg/dl, HbA1c < 6.5% and no history of T2DM. According to the ADA criteria [23], individuals were classified as having normal glucose tolerance (NGT) when fasting plasma glucose was < 100 mg/dl (5.5 mmol/l), 2-h postload glucose < 140 mg/dl (< 7.77 mmol/l) and HbA1c < 5.7%, prediabetes when fasting plasma glucose was 100–125 mg/dl (5.5–6.9 mmol/l), 2-h postload glucose 140–199 mg/dl (7.77–11.0 mmol/l) or HbA1c 5.7–6.4%, drug-naïve newly diagnosed T2DM when fasting plasma glucose was ≥ 126 mg/dl (> 7 mmol/l), 2-h post-load glucose was ≥ 200 mg/dl (> 11.1 mmol/l), HbA1c ≥ 6.5%. The HOMA-IR index was calculated as fasting insulin × fasting glucose/22.5 [25].

The study was approved by the Ethical Committee (Comitato Etico Azienda Ospedaliera “Mater Domini”), and informed consent was obtained from each subject in accordance with principles of the Declaration of Helsinki.

### Echocardiographic measurements

Tracings were taken with participants in a partial left decubitus position using a VIVID-7 Pro ultrasound machine (GE Technologies, Milwaukee, WI, USA) with an annular phased array 2.5-MHz transducer. All the readings were performed by the same experienced investigator to optimize the reproducibility, blinded to the clinical data of the examined individuals. Tracings were recorded under two-dimensional guidance, and M-mode measurements were taken at the tip of the mitral valve or just below. Measurements of interventricular septum thickness (IVS), posterior wall thickness (PWT) were made at end-diastole. LV end-diastolic (LVEDV) and end-systolic volume (LVESV) were measured according to Simpson method and indexed for body surface area (BSA) [26]. LV mass (LVM) was calculated using the Devereux formula [27] and normalized by BSA [LVMI]) [26, 28].

### Myocardial mechano-energetic efficiency measurements

The myocardial mechano-energetic efficiency (MEE) can be defined as the ratio between the external systolic work, and the amount of total energy produced for each contraction [21, 22, 29–31]. External myocardial work can be estimated as stroke work (SW), with SW being computed as systolic blood pressure (SBP) x echocardiographic stroke volume (SV). SV was calculated as the difference between LV end-diastolic and end-systolic volumes by the z-derived method, and allometrically normalized by height (Stroke Index) [29–31]. Myocardial oxygen consumption (MVO_2_) reflects the total amount of energy produced by the myocardium, and can be estimated using the “double product” (DP) of SBP x heart rate (HR) [32]. Thus, MEE may be estimated as: SBP × SV/ SBP × HR = SV/HR.

where HR were expressed in seconds (HR/60). Because MEE is highly related to LV mass, MEE was normalized for LV mass with the purpose of obtaining an estimate of energetic expenditure per unit of myocardial mass (i.e. indexed MEE, MEEi, ml/s/g) [21, 22, 29–31].

### Laboratory determinations

Plasma glucose, total and HDL cholesterol, and triglycerides were assayed using enzymatic methods (Roche Diagnostics, Mannheim, Germany). HbA1c was measured with high performance liquid chromatography using an NGSP-certified automated analyzer (Adams HA-8160 HbA1c analyzer, Menarini, Italy). Plasma insulin concentration was determined with a chemiluminescence-based assay (Immulite, Siemens, Italy).

### Statistical analyses

Variables with skewed distribution including fasting insulin, and triglycerides were natural log transformed for statistical analyses. Continuous variables are expressed as means ± SD. Categorical variables were compared by χ^2^ test. Comparisons between women and men were performed using unpaired Student’s t test. Comparisons between NGT, prediabetes and newly diagnosed T2DM groups were performed separately in men and women using a general linear model with post hoc Fisher's least significant difference correction for pairwise comparisons. For all analyses a P value < 0.05 was considered to be statistically significant. All analyses were performed using SPSS software Version 22 for Windows.

## Results

### Cardiovascular risk factors in subjects with NGT, prediabetes and newly diagnosed T2DM according to sex

Anthropometric and cardiovascular features of individuals with NGT, prediabetes and drug-naïve newly diagnosed T2DM according to sex are shown in Table 1. No sex-related differences in age were observed across the three glucose tolerance categories. In NGT group, men were more probable to be current smokers, and exhibited significantly higher abdominal adiposity as measured by waist circumference than women, and had significantly higher levels of systolic (SBP) and diastolic blood pressure (DBP), triglycerides, FPG, fasting insulin, HOMA-IR, and lower levels of HDL cholesterol (Table 1). However, women with NGT showed higher levels of heart rate than men (Table 1).

**Table 1:** Differences in clinical characteristics of men and women with NGT, prediabetes, and newly diagnosed T2DM

	Women			Prediabetes vs. NGT	T2DM vs. NGT	Men			Prediabetes vs. NGT	T2DM vs. NGT	Women vs. men (NGT)	Women vs. men (prediabetes)	Women vs. men (T2DM)
	NGT (n = 456)	Prediabetes (n = 274)	T2DM (n = 74)	P value^§^	P value^§^	NGT (n = 314)	Prediabetes (n = 321)	T2DM (n = 123)	P value^§^	P value^§^	P values	P value	P value
Age (yrs)	50 ± 8	54 ± 8	57 ± 8	< 0.0001	< 0.0001	49 ± 8	54 ± 8	55 ± 8	< 0.0001	< 0.0001	0.2	0.85	0.08
BMI (kg/m^2^)	28.8 ± 5.6	30.7 ± 5.5	32 ± 5.2	< 0.0001	< 0.0001	29.1 ± 4.5	30.1 ± 4.8	30 ± 4.7	0.005	0.01	0.38	0.16	0.01
Waist circumference (cm)	96.7 ± 13	101.7 ± 13	106.3 ± 12	< 0.0001	< 0.0001	102 ± 11	105 ± 12	104 ± 11	0.009	0.06	< 0.0001	0.005	0.21
Smoking status (never smokers/current smokers/ex-smokers) (%)	69/18/13	72/13/15	70/11/19	< 0.0001	< 0.0001	35/33/32	37/23/40	28/25/47	< 0.0001	0.06	< 0.0001	0.001	0.07
Systolic blood pressure (mmHg)	127 ± 17	130 ± 17	138 ± 18	0.21	0.002	132 ± 15	136 ± 16	135 ± 16	0.01	0.18	< 0.0001	< 0.0001	0.27
Diastolic blood pressure (mmHg)	78 ± 11	80 ± 10	81 ± 11	0.06	0.45	84 ± 10	83 ± 10	83 ± 10	0.89	0.69	< 0.0001	0.001	0.14
Heart rate (beats min^−1^)	70 ± 8	72 ± 10	72 ± 11	0.01	0.01	69 ± 10	70 ± 10	72 ± 10	0.38	0.002	0.04	0.01	0.93
Total cholesterol (mg/dl)	203 ± 39	214 ± 42	201 ± 39	0.006	0.21	204 ± 41	202 ± 38	196 ± 44	0.53	0.02	0.74	< 0.0001	0.38
HDL (mg/dl)	58 ± 13	54 ± 12	50 ± 15	< 0.0001	< 0.0001	45 ± 11	45 ± 11	43 ± 12	0.43	0.06	< 0.0001	< 0.0001	0.002
Triglycerides (mg/dl)	107 ± 59	133 ± 66	164 ± 107	< 0.0001	< 0.0001	143 ± 86	148 ± 78	164 ± 126	0.4	0.057	< 0.0001	0.01	0.99
Fasting Glucose (mg/dL)	87 ± 7	99 ± 11	133 ± 58	< 0.0001	< 0.0001	90 ± 6	103 ± 9	138 ± 51	< 0.0001	< 0.0001	< 0.0001	< 0.0001	0.54
2-h glucose (mg/dl)	105 ± 20	147 ± 28	230 ± 32	< 0.0001	< 0.0001	105 ± 20	139 ± 32	227 ± 45	< 0.0001	< 0.0001	0.92	0.001	0.7
HbA1c (%)	5.3 ± 0.3	5.6 ± 0.3	7.07 ± 1.5	< 0.0001	< 0.0001	5.4 ± 0.3	5.7 ± 0.3	7.02 ± 1.5	< 0.0001	< 0.0001	0.3	0.17	0.86
Fasting Insulin (µU/ml)	11 ± 6	14 ± 7	18 ± 12	< 0.0001	< 0.0001	13 ± 7	14 ± 8	16 ± 10	< 0.0001	< 0.0001	0.001	0.62	0.17
HOMA-IR	2.38 ± 1.3	3.5 ± 1.9	5.4 ± 4.8	< 0.0001	< 0.0001	2.8 ± 1.7	3.7 ± 2.1	5.5 ± 4.4	< 0.0001	< 0.0001	< 0.0001	0.27	0.55
Antihypertensive therapy (%)	61.3	54.3	58	0.13	0.57	56.7	47.6	56	0.06	0.93	0.21	0.1	0.79
Lipid-lowering therapy (%)	13.5	16.4	17.4	0.54	0.26	14	11.2	14.7	0.28	0.41	0.7	0.06	0.45

Data are means ± SD, unless otherwise indicated. Categorical variables were compared by χ2 test. Comparisons between the three groups of glucose tolerance were performed using a general linear model with post hoc Fisher's least significant difference correction for pairwise comparisons. Comparisons between women and men were performed using unpaired Student’s t test. ^§^P values refer to results after analyses with adjustment for age. Insulin, and triglyceride levels were log transformed for statistical analysis, but values in the table represent a back transformation to the original scale

Among prediabetic subjects, men were more probable to be current smokers, and exhibited significantly higher values of abdominal adiposity, blood pressure, triglycerides, FPG, and lower levels of HDL cholesterol than women. However, prediabetic women showed higher levels of heart rate, total cholesterol, and 2-h post-load glucose than men.

Among newly diagnosed T2DM patients, women were heavier, and showed higher levels of HDL cholesterol than men. No sex-related differences in the proportion of individuals treated with antihypertensive or lipid-lowering therapies were observed across the three glucose tolerance categories.

### Age-adjusted differences in cardiovascular risk factors between women with NGT, prediabetes and newly diagnosed T2DM

As shown in Table 1, worsening of glucose tolerance from NGT to prediabetes to newly diagnosed T2DM in women was associated with a progressive increase in age-adjusted values of BMI, waist circumference, heart rate, triglycerides, fasting insulin, HOMA-IR, and a decrease in HDL cholesterol. Additionally, as compared with women with NGT, those with newly diagnosed T2DM showed a significant increase in SBP (Table 1). By contrast, women with NGT were more probable to be current smokers than prediabetic and newly diagnosed T2DM women. No differences in the proportion of individuals treated with antihypertensive or lipid-lowering therapies were observed across the three glucose tolerance categories.

### Age-adjusted differences in cardiovascular risk factors between men with NGT, prediabetes and newly diagnosed T2DM

As compared with men with NGT, both prediabetic and newly diagnosed T2DM men were heavier, and showed an increase in age-adjusted fasting insulin levels, and HOMA-IR (Table 1). In addition, as compared with men with NGT, those with prediabetes were less probable to be current smokers, and showed a significant increase in SBP, while newly diagnosed T2DM individuals exhibited higher heart rate, and lower total cholesterol levels (Table 1).

**Fig. 1:**
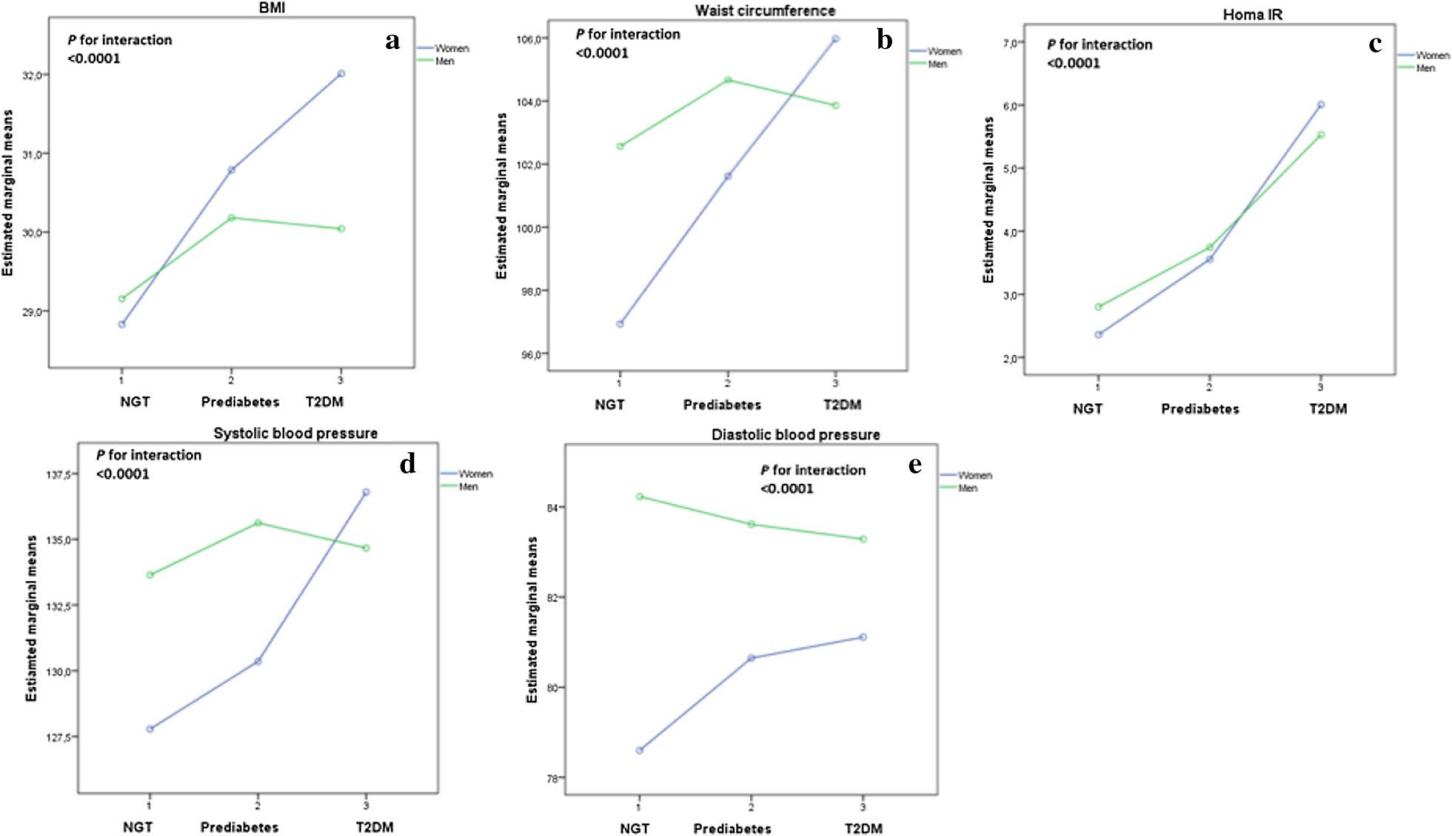
The estimated marginal means of cardiovascular variables adjusted for age according to sex and glucose tolerance status. **a** BMI; **b** waist circumference; **c** HOMA-IR index; **d** systolic blood pressure; and **e** diastolic blood pressure

The estimated marginal means of cardiovascular variables adjusted for age according to sex and glucose tolerance status are reported in Fig. 1. Prediabetic and newly diagnosed T2DM women exhibited greater relative differences in BMI, waist circumference, blood pressure, and HOMA-IR, than prediabetic and diabetic men when compared with their NGT counterparts. Formal tests for glucose tolerance status × sex interaction were statistically significant for BMI (P < 0.0001), waist circumference (P < 0.0001), blood pressure (P < 0.0001), and HOMA-IR (P < 0.0001) (Fig. 1).

### Left ventricular mass, and mechano-energetic efficiency in subjects with NGT, prediabetes and newly diagnosed T2DM according to sex

As shown in Table 2, worsening of glucose tolerance from NGT to prediabetes to newly diagnosed T2DM in women was associated with a progressive increase in LVMI, and a decrease in myocardial MEEi after adjustment for age, smoking status, and antihypertensive therapy. Prediabetic and newly diagnosed diabetic men exhibited an increase in LVMI, and a decrease in myocardial MEEi as compared with NGT men after adjustment for age, smoking status, and antihypertensive therapy (Table 2).

**Table 2:** Differences in echocardiographic and mechano-energetic efficiency parameters in men and women with NGT, prediabetes and newly diagnosed T2DM

Echocardiographic variables	Women			Prediabetes vs. NGT	T2DM vs. NGT	Men			Prediabetes vs. NGT	T2DM vs. NGT	Women vs. men (NGT)	Women vs. men (prediabetes)	Women vs. men (T2DM)
	NGT(n = 456)	Prediabetes (n = 274)	T2DM (n = 74)	P value§	P value§	NGT (n = 314)	Prediabetes (n = 321)	T2DM (n = 123)	P value§	P value§	P values	P value	P value
LV end-systolic volume (LVESV) (*ml*)	29.1 ± 11	31 ± 17	31.3 ± 13	0.1	0.32	38.9 ± 19	41.7 ± 23	42.3 ± 24	0.24	0.21	< 0.0001	< 0.0001	< 0.0001
LV end-diastolic volume (LVEDV) (*ml*)	107.5 ± 28	108 ± 33	111 ± 32	0.77	0.38	135.4 ± 40	137.5 ± 47	134.8 ± 41	0.65	0.72	< 0.0001	< 0.0001	0.02
Interventricular septal thickness (IVS) (*cm*)	1.00 ± 0.2	1.05 ± 0.16	1.12 ± 0.16	0.07	0.003	1.13 ± 0.2	1.17 ± 0.15	1.17 ± 0.17	0.13	0.47	< 0.0001	< 0.0001	0.001
Posterior wall thickness (PWT) (*cm*)	0.85 ± 0.14	0.89 ± 0.16	0.91 ± 0.15	0.02	0.04	0.93 ± 0.14	0.96 ± 0.14	0.98 ± 0.15	0.04	< 0.0001	< 0.0001	< 0.0001	0.001
LV mass index (LVMI) (*g/m*^*2*^)	95.8 ± 24	101.7 ± 26	114 ± 34	0.12	0.001	113.1 ± 27	120.7 ± 31	123.2 ± 33	0.08	0.01	< 0.0001	< 0.0001	0.06
Myocardial MEEi (ml/s g^−1^)	0.41 ± 0.12	0.36 ± 0.10	0.34 ± 0.08	< 0.0001	< 0.0001	0.38 ± 0.11	0.35 ± 0.09	0.33 ± 0.1	0.005	< 0.0001	0.001	0.08	0.39

Data are means ± SD, unless otherwise indicated. Comparisons between women and men were performed using unpaired Student’s t test. Comparisons between the three groups of glucose tolerance were performed using a general linear model with post hoc Fisher's least significant difference correction for pairwise comparisons. ^§^P values refer to results after analyses with adjustment for age, smoking status, and antihypertensive therapy

Amongst NGT subjects, men displayed significantly higher levels of LVEDV, LVESV, IVS, PWT, and LVMI than women, while myocardial MEEi was lower in men as compared with women after adjustment for age (Table 2). Among prediabetic subjects, men exhibited significantly higher values of LVEDV, LVESV, IVS, PWT, and LVMI as compared with women after adjustment for age (Table 2), but no sex-specific differences were observed in myocardial MEEi. No statistically significant differences were observed in LVMI, and myocardial MEEI between men and women with newly diagnosed T2DM after adjustment for age (Table 2).

**Fig. 2:**
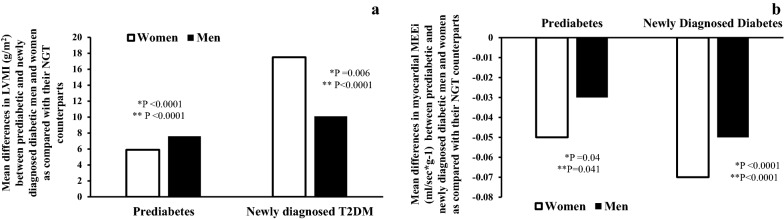
**a** Mean differences in LVMI (g/m^2^) between prediabetic and newly diagnosed diabetic men and women as compared with their NGT counterparts; **b** Mean differences in myocardial MEEi (ml/s g^−1^) between prediabetic and newly diagnosed diabetic men and women as compared with their NGT counterparts. *P values refer to results after analyses with adjustment for age. ** P values refer to results after analyses with adjustment for age, smoking status, and antihypertensive therapy

As shown in Fig. 2a, prediabetic men showed greater relative differences in LVMI than prediabetic women as compared with their NGT counterparts after adjustment for age. These differences in LVMI remained significant after further adjustment for smoking status, and antihypertensive therapy. By contrast, newly diagnosed diabetic women showed greater relative differences in LVMI than newly diagnosed diabetic men as compared with their NGT counterparts after adjustment for age. These differences in LVMI remained significant after further adjustment for smoking status, and antihypertensive therapy.

As shown in Fig. 2b, both prediabetic and newly diagnosed diabetic women showed greater relative differences in myocardial MEEi than prediabetic and newly diagnosed diabetic men as compared with their NGT counterparts after adjustment for age. These differences in myocardial MEEi than prediabetic and newly diagnosed diabetic men as compared with their NGT counterparts remained significant after further adjustment for smoking status, and antihypertensive therapy.

**Fig. 3:**
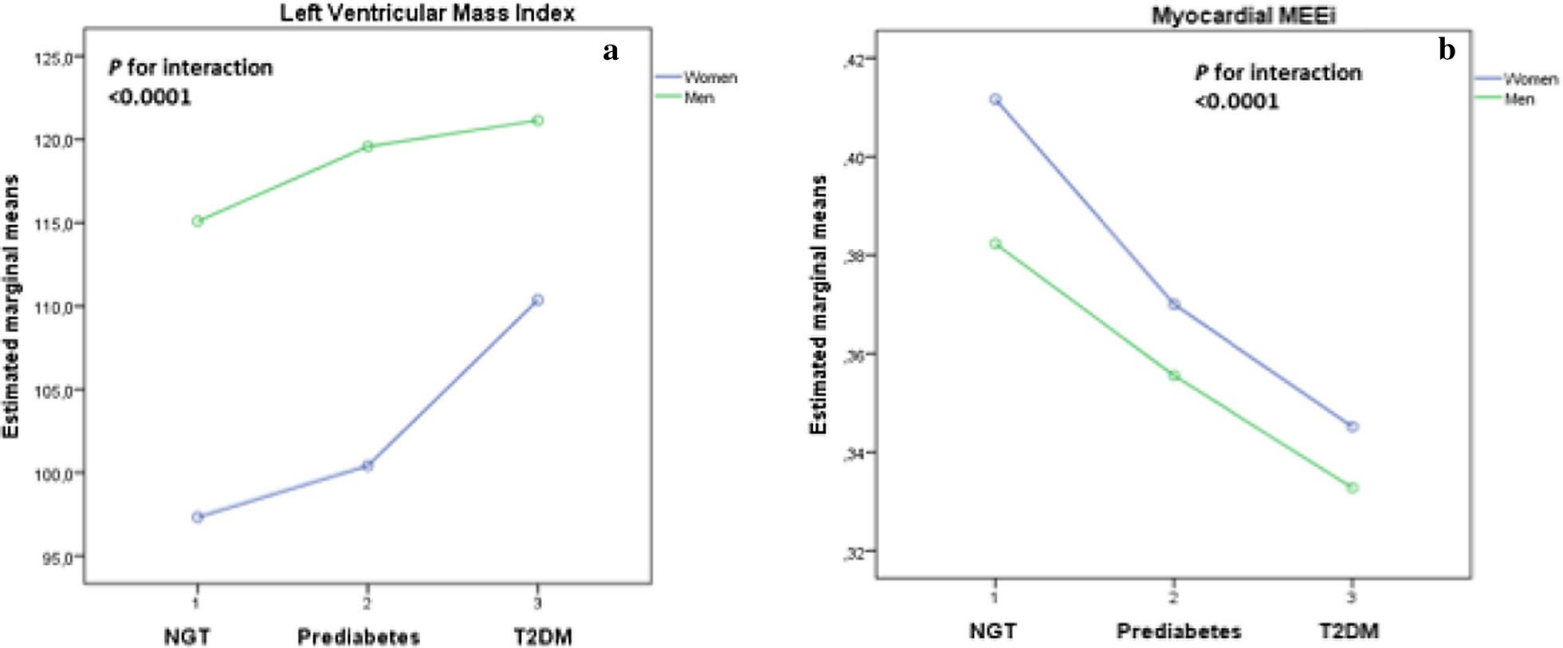
The estimated marginal means of cardiovascular variables adjusted for age according to sex and glucose tolerance status. **a** LVMI (g/m^2^); **b** myocardial MEEi (ml/s g^−1^)

The estimated marginal means of LVMI and myocardial MEEi adjusted for age according to sex and glucose tolerance status are shown in Fig. 3. Formal tests for glucose tolerance status × sex interaction were statistically significant for myocardial MEEi (P < 0.0001), and LVMI (P < 0.0001), respectively.

## Discussion

Left ventricular hypertrophy is a well-established organ damage causing adverse metabolic, functional, and structural cardiac changes, which ultimately lead to unfavourable cardiovascular outcomes [20]. An increase in left ventricular mass (LVM) is associated with enhanced myocardial oxygen consumption thus increasing the risk of myocardial infarction. Cardiac metabolism predominantly relies on aerobic oxidation of substrate for energy generation with close coupling between myocardial oxygen consumption and LV function [33–35]. Under physiological conditions, the proportion of energy produced by the heart which is used for contraction is about 25%, and the residual energy is chiefly dissipated as heat. Thus, mechanical energetic efficiency of the heart can be defined as the ratio of external work performed by the left ventricle (measurable as stroke work i.e. SBP x stroke volume) and the amount of oxygen consumed during contraction [33–35]. Although cardiac energy consumption can precisely be measured invasively by coronary sinus catheterization [33] or noninvasively by positron emission tomography [36], both measurements are not feasible in routine clinic evaluation. To overcome these problems, a simple non-invasive, ultrasound-based method estimating myocardial mechano-energetic efficiency per gram of left ventricular mass (MEEi) has been developed and validated [21, 22, 30–32]. In this cross-sectional study aimed at exploring sex-related differences in LVM, and myocardial MEEi across glucose tolerance conditions, we found that worsening of glucose tolerance from NGT to prediabetes to drug-naïve newly diagnosed T2DM in both men and women was associated with a progressive increase in LVMI and myocardial MEEi after adjustment for potential confounders including age, smoking status, and antihypertensive therapy. More importantly, we found that women with drug-naïve newly diagnosed T2DM exhibited greater relative differences in LVMI and myocardial MEEi than men with newly diagnosed T2DM when compared with their NGT counterparts after adjustment for age, smoking status, and antihypertensive therapy (Fig. 2, and Table 2). Moreover, we found that prediabetic women exhibited greater relative differences in myocardial MEEi, but not in LVMI, than prediabetic men when compared with their NGT counterparts after adjustment for age, smoking status, and antihypertensive therapy (Fig. 2, and Table 2). Although NGT men exhibited significantly higher LVMI and lower myocardial MEEi than NGT women, these differences were markedly diminished when comparing prediabetic men and women and were abolished comparing men and women with newly diagnosed T2DM. Notably, the statistical test for interaction between sex and glucose tolerance on both LVMI and myocardial MEEi was statistically significant (Fig. 3) thus suggesting the existence of a sex-specific association. These results are in agreement with previous studies on sex-specific differences in the association between glucose tolerance and LVMI [37–41], and also extend previous investigations as we are the first to show sex-related differences in the association between glucose tolerance and myocardial MEEi. Overall, the present findings suggest that the impact of the hyperglycemic burden on cardiac organ damage such as increased LVM and decreased myocardial MEEi is stronger in women than in men after exceeding the diagnostic threshold of T2DM. However, prediabetic women did not yet manifest echocardiographic evidence of increased LVM observed in prediabetic men suggesting that more pronounced alteration in glucose metabolism are necessary to increase LVM. By contrast, the moderately elevated levels of glycemia typically associated with the prediabetic condition are sufficient to affect to a greater extent myocardial MEEi in women than in men (Fig. 2, and Table 2).

The pathophysiological mechanisms underpinning the excess cardiac organ damage conferred by hyperglycemia in women compared with men remain speculative. However, there are some plausible candidates that may explain this phenomenon. Several prior investigations have demonstrated that the differences in cardiovascular risk factors associated with deterioration of glucose tolerance are greater in women than men [9–11, 40, 41]. Accordingly, we found that prediabetic and newly diagnosed T2DM women exhibited greater relative differences in BMI, waist circumference, blood pressure, and HOMA-IR than prediabetic and men with newly diagnosed T2DM when compared with their NGT counterparts with formal tests for glucose tolerance status × sex interaction being statistically significant (Table 1 and Fig. 1). However, the relevance of these differences in cardiovascular risk factors on cardiac organ damage should be interpreted in light of a prior study showing that a 2 years multi-intervention including lifestyle intervention and pharmacologic treatment to reach strict cardiovascular risk factor goals in patients with T2DM had neutral impact on systolic and diastolic cardiac function [42]. Obesity and hypertension are two pathophysiological factors involved in the development of left ventricular hypertrophy [43], and sex-specific differences in the geometric adaptations of the left ventricle to the coexistence of obesity and hypertension have been reported with women with concurrent obesity and hypertension exhibiting higher increase in the prevalence of left ventricular hypertrophy than men [44]. Indeed, it has been reported that myocardial volume increased with higher age and BSA, with an additional gender dependency both gender and body surface area were associated with left ventricle volumes, and myocardial volume [45]. Moreover, it has been shown that increased levels of adipocyte fatty acid-binding protein (AFABP), a lipid chaperone protein linked to obesity, is associated with a significant longitudinal increase in left ventricular mass, and is an independent predictor of incident major adverse cardiovascular events in patients with T2DM [46].

There is evidence that treatments with metformin or statins impact myocardial metabolism in a sex-specific fashion thus raising the possibility that the observed results might be due to differences in the prescribed therapeutic regimens [47]*.* However, none of the participants was treated with metformin, and no sex-related differences were found in the treatment with anti-hypertensive, and lipid-lowering medications to manage cardiovascular risk factors in individuals with prediabetes or drug-naïve newly diagnosed T2DM thus arguing against the possibility that prediabetic diabetic women receive less cardiovascular-risk-modifying therapies compared to their male counterparts.

Insulin resistance is another pathophysiological factor involved in the development of left ventricular hypertrophy, and impairment in myocardial MEEi even in the presence of obesity and hypertension [17, 30, 48–50]. We found that levels of insulin resistance, assessed as HOMA-IR index, differed more between women with prediabetes and newly diagnosed diabetes than between their male counterparts (Table 1 and Fig. 1). Notably, although NGT women exhibited higher insulin sensitivity than NGT men, prediabetic and newly diagnosed T2DM women have the same degree of insulin resistance than their male counterparts. Our results are in agreement with a recent study showing significant abnormalities in myocardial deformation in obese adolescents with dysglycemia and insulin resistance compared with their lean normoglycemic counterparts [51]. Insulin resistance affects LVM and myocardial MEEi by various mechanisms. There is evidence that in patients with T2DM, myocardial steatosis, a marker of insulin resistance, is independently associated with myocardial concentric remodeling [52]. Moreover, insulin resistance induces a shift of cardiac metabolism towards free fatty acid oxidation at the expense of glucose, leading to an increased myocardial oxygen consumption and decrease of myocardial energetic efficiency [21, 24, 30, 48, 49]. Moreover, insulin resistance is associated with activation of renin–angiotensin–aldosterone system (RAAS) thus promoting the stimulating effects of angiotensin II on cellular growth and collagen production, which leads to myocardial hypertrophy and fibrosis [49, 53]. Furthermore, insulin resistance is associated with endothelial dysfunction that may contribute to increase LVM by loss of its modulating role in the synthesis of extracellular matrix components and by shifting the local myocardial homeostasis toward hypertrophy [49].

An addition pathophysiological factor that might have an impact on left cardiac structure in individuals with pre-diabetes and T2DM is glycemic variability. It has been reported that visit‑to‑visit fasting plasma glucose variability is associated subclinical left cardiac remodeling and systolic dysfunction, independently of conventional risk factors [54]. Additionally, evidence has been provided that glycemic gap, a marker of glycemic excursion that measures the magnitude of a relative glycemic rise from chronic glycaemia, was associated with a change in left ventricular ejection fraction, and a high risk of postinfarct left ventricular systolic dysfunction [55].

This study has some strengths: (1) the relatively large size of the well-characterized CATAMERI cohort; (2) the precise assessment of glucose tolerance by FPG, 2 h post-load glucose levels during an OGTT, and HbA1c according to ADA criteria to exclude any potential misclassification of participants [23]; (3) the availability of clinical and prescribing information of people with and without T2DM; (4) the exclusion of individuals treated with glucose-lowering agents including metformin or with drugs known to affect glucose metabolism such as corticosteroids and estroprogestins used for hormonal contraception or replacement treatment; (5) the homogeneous ethnic background that exclude the possibility that wide genetic variation might affect sex-associated differences in organ damage; and (6) the echocardiographic measurements made by an experienced examiner who was blinded to the clinical data of the study participants.

Nonetheless, this study has also some limitations. Myocardial mechano-energetic efficiency was estimated by indirect measures rather than by coronary sinus catheterization [33] or by cardiac positron emission tomography [36]. However, these measurements are invasive, expensive, and time-consuming thus making these procedures not feasible in clinical practice, and epidemiological studies. Moreover, this analysis of the CATAMERI cohort study includes only Caucasian individuals aging between 40 and 70 years with at least one cardiovascular risk factors attending a referral university hospital, thus limiting the generalizability of the present results to other ethnicities or to white Caucasians cohorts. Although statistical analyses were adjusted for several covariates, residual confounders such as socio-economic status may have affected the results. Additionally, the cross-sectional design and the observational nature of this study do not permit any causal inferences. Third, since our study population was composed by individuals with at least one cardiovascular risk factors attending a referral university hospital, our findings may not be extendible to the general population.

## Conclusions

The current study suggests that left ventricle is subject to maladaptive changes involving left ventricular mass and myocardial mechanical energetic efficiency with worsening of glucose tolerance, especially in women with newly diagnosed T2DM. The sex-specific increase in LVM and decrease in MEEi, both being predictors of cardiovascular events [20–22], may contribute to explain, at least in part, the stronger impact of T2DM on the excess risk of cardiovascular disease in women than in men. Overall, these data highlight the importance of greater awareness of sex-related differences in cardiovascular organ damage in subjects with early impairment of glucose homeostasis in order to promote appropriate lifestyle change intervention and, ultimately, pharmacological treatments.

## Data Availability

The datasets used and analysed during the current study are available from the corresponding author on reasonable request.

## Abbreviations

T2DM: Type 2 diabetes mellitus
RR: Relative risk
CVD: Cardiovascular disease
OGTT: Oral glucose tolerance test
NGT: Normal glucose tolerance
ADA: American Diabetes Association
FPG: Fasting plasma glucose
BMI: Body mass index
IVS: Interventricular septum thickness
PWT: Posterior wall thickness
LVEDV: Left ventricular end-diastolic volume
LVESV: Left ventricular end-systolic volume
BSA: Body surface area
LVM: LV mass
LVMI: LV mass normalized by BSA
MEE: Mechano-energetic efficiency
SW: Stroke work
SBP: Systolic blood pressure
SV: Stroke volume
SI: Stroke volume normalized by height
MVO_2_: Myocardial oxygen consumption
DP: Double product
HR: Heart rate
MEEi: Mechano-energetic efficiency indexed
DBP: Diastolic blood pressure
RAAS: Renin–angiotensin–aldosterone system
